# Grief in Times of Corona (Envoi)

**DOI:** 10.1177/1077800420960140

**Published:** 2021-09

**Authors:** Cathy Fowley

**Affiliations:** 1 Independent Scholar, Dublin, Ireland

**Keywords:** chronotope, poetry, autoethnography, COVID

## Abstract

This autoethnographic poem tells of personal grief happening in a time of lockdown. It draws on the concept of chronotope, a discrete time and space unit, a parenthesis of sorts, which I have chosen to illustrate as a bubble. In our daily speech, we see bubbles as related to both time and space, now with the added meaning of close relationship of people, those who belong to the same COVID bubble. In this autoethnographic piece, relationships are mediated by technology which anchors our bubbles together, with multimodal links carrying affect and emotion.

This autoethnographic piece was written in secluded place in the West of Ireland, in what everyone called unprecedented times, and at a time when space became both fragmented and legally bound throughout the world. It was the outcome of my participation in the massive and microscopic sensemaking project (Markham et al., 2020), inspired by some of the prompts, nourished by the work of other writers and visual artists.

Grief catches me in hidden places. I am haunted by chronotopes,^1^ I see them everywhere. A time and place outside time and place, lifetime, the time of a story, a beginning, a middle, and an end.

In my garden, two small girls play hula hoop and blow bubbles.

Bubbles within bubbles, constraining place, constraining time. Bubbles within bubbles within bubbles, in a world which has stepped outside its narrative arc. To have story, I say in my classes, you need to start with “one day.”^2^

One day, all the doors closed. Time and place became bound by decree.

Three weeks later, she would spend the day in pain, in hospital, having heard the previous week, all of a sudden (one day, this happened) that she had Stage 4 pancreatic cancer. Her sterilized bubble was 300 km away from my green bubble. Where do stories start, where do they end? And how do bubbles pop inside other bubbles?

**Bubble one—WhatsApp, tendrils of love**

We don’t speak, voices break, words disappear.

We send images,

technological tendrils of hope and love.

I walk, as she walked;

I take photographs on my phone, of roads, bridges, lake,

my 2 km bubble.

*  A video of the movement of a swing*

***love the swing***

***and all it represents.***

  *In the golden evening light*,

  *a bridge of fallen logs, a few words*

***Emoticon: a heart***

From a darkened bedroom, two hospital rooms, her veranda, another hospital room,she takes photographs of windows.

Frames and grids,

outside is bright, inside darker.

*I think of a poem in Joan McBreen’s Maps and Atlas*^3^

  *[large windows would frame*

  *A world without, bring it in*,

  *Allow its light over our stories]*

I pick wild roses and take a photograph.

The two grey ticks never turn blue.

**Bubble two—numbers**

25,489 cases in Ireland

1,738 deaths

112 days since I saw her

47 days since I heard her voice.

**Bubble three—helicopter, tendrils of fear**

I walk within a circle, two kilometres drawn on an app,

circling space, allowed space, cutting it out, shading it.

I walk in a time bubble, a now unmoored from the before and the after.

A sound creates a circle, fear and anxiety layered

A small boy lost in the lake,

The sound of the helicopter that flies and hovers

Looking for a body,

A sharp pain

Echoing the sound of

Helicopters landing on

The roofs of hospitals

Can she hear them

Echoing the sound of

Helicopters circling

Watching

Malevolent

Over crowds in cities

Far away.^4^

This is death announced

By sound and

Machine.

**Bubble four—tendrils of grief**

I embroider circles

I don’t read the news

I retreat into my small bubble

Shrink it to its limit.

She couldn’t breathe on her own any more,and when I hear her husband’s voice on thephone I too stop breathing

for one piercing instant.

## Notes

1. Bakhtin writes about the literary artistic chronotope, where “spatial and temporal indicators are fused into one carefully thought-out, concrete whole. Time, as it were, thickens, takes on flesh, becomes artistically visible; likewise, space becomes charged and responsive to the movements of time, plot and history” (Bakhtin, 1981, p. 84).

2. I talk about story, about narrative. Bakhtin talks about the novel when he writes that “‘suddenly’ and ‘at just that moment’ best characterizes that type of time, for this time usually has its origin and comes into its own in just those places where the norms, pragmatic and premeditated course of events is interrupted” (Bakhtin, 1981, p. 92). And sometimes there is very little difference between our lives and the narrative of our lives.

3. The poem Blackberry Time on the High Road holds those lines that echoed in my mind as I read the messages and saw the photographs of windows, far from the joyous family home in McBreen’s poem (McBreen, 2017, p. 19).

4. In the co-authored introduction to the *Handbook of Autoethnography*, Carolyn Ellis writes that “what autoethnography is teaching me today is this: telling our stories is a way for us to be present to each other, provides a space for us to create a relationship embodied in the performance of writing and reading what is reflective, critical, loving, and chosen in solidarity” (Holman Jones et al., 2016, p. 19).
